# Engineering Microstructure-Sensitized Paper-Based Flexible Tactile Sensor with Wide Pressure Range and High Sensitivity

**DOI:** 10.3390/mi17080948

**Published:** 2026-08-09

**Authors:** Hongyu Yao, Hongyun He, Qingxu Zheng, Ruizhi Peng, Wenxiang Hu, Duo Chen

**Affiliations:** Faculty of Optoelectronic Science and Technology, Qilu University of Technology (Shandong Academy of Sciences), Jinan 250353, China; yaohongyu1028@163.com (H.Y.); 202486033073@stu.qlu.edu.cn (H.H.); 202486033014@stu.qlu.edu.cn (Q.Z.); 202486033069@stu.qlu.edu.cn (R.P.); 202486033071@stu.qlu.edu.cn (W.H.)

**Keywords:** flexible tactile sensor, sensitization, toilet paper/AgNWs, wide pressure range, high sensitivity

## Abstract

With the widespread adoption of the Internet of Things and wearable technology, flexible tactile sensors—serving as core components for detecting external mechanical signals—have become a key supporting technology across numerous fields. Piezoresistive flexible tactile sensors offer advantages such as simple structure, high sensitivity, and ease of integration. Paper-based sensing materials sensitized with nanomaterials are simple to prepare and low-cost, making them suitable candidates for tactile sensor fabrication. However, paper-based tactile sensors typically cannot simultaneously achieve a wide detection range and high sensitivity. This paper presents an engineered microstructure-sensitized flexible tactile sensor based on toilet paper/silver nanowires (AgNWs). This study integrates the structural advantages of engineered polydimethylsiloxane (PDMS) microstructures with the synergistic effects of toilet paper/silver nanowires (AgNWs) to construct a high-performance flexible sensing system. The device exhibits a wide pressure detection range (6.85–273.96 kPa), high sensitivity (39,570 kPa^−1^), response and recovery times on the order of hundreds of milliseconds, and stable operation over approximately 10,000 cycles. This sensor demonstrates promising application potential in wearable biosensing, health monitoring, and related fields.

## 1. Introduction

Flexible tactile sensors, characterized by fast response speed, flexibility, and biocompatibility, hold potential in applications such as wearable electronics, electronic skin, health monitoring, and intelligent robotics, thus attracting extensive research attention from the academic community [1,2,3]. Recent advances in multimodal sensing textiles have further expanded the application scope of flexible wearable devices [4].

The core function of flexible tactile sensors is to convert mechanical forces applied to piezoresistive materials into electrical signals. Depending on their sensing mechanisms, these sensors are primarily categorized into three types: resistive, capacitive, and piezoelectric. Resistive sensors detect changes in resistance between flexible electrodes and force-sensitive materials; capacitive sensors measure capacitance fluctuations caused by variations in the plate separation of parallel-plate capacitors; and piezoelectric sensors directly convert mechanical energy into electrical energy through the polarization effect of piezoelectric materials. Among these, resistive flexible sensors have attracted extensive attention in academia due to their simple fabrication process, strong robustness, and stability.

Sensitivity is a key performance indicator of flexible pressure sensors, directly determining their operational efficiency and measurement accuracy. Current approaches to enhancing sensitivity mainly fall into two categories: one involves introducing micro- and nanostructures such as pyramids, three-dimensional hollow spheres, and porous sponges, which exhibit greater deformability compared to conventional structures and thus effectively improve measurement precision; the other employs nanomaterials like graphene [5,6], carbon nanotubes [7], metallic nanowires [8], and polymer fibers [9] for surface modification and enhancement.

Among various polymer substrates, elastomers represented by polydimethylsiloxane (PDMS) are regarded as suitable candidates for flexible sensors due to their intrinsic flexibility, low elastic modulus, favorable machinability, chemical stability and biocompatibility. PDMS can replicate sophisticated microstructures (e.g., micro-pyramids) to effectively amplify mechanical deformation, which further improves the sensing performance [10]. For active nanoscale sensing materials, laser-induced graphene (LIG) enables customizable patterning via direct laser writing, yet it relies on specialized fabrication equipment and exhibits limited sensitivity under low-pressure regimes [11]. MXene possesses ultrahigh electrical conductivity but suffers from severe oxidative degradation and poor long-term operational stability [12]. Carbon nanotubes (CNTs) display mechanical properties, while their unsatisfactory dispersibility and high inter-tube contact resistance inevitably increase the baseline resistance and restrict the sensing performance. In comparison, silver nanowires (AgNWs) exhibit advantages including high conductivity, low percolation threshold, flexibility and mature liquid-phase synthesis technology. AgNWs possess low wire-to-wire contact resistance, form stable bonding with paper fibers, and can be effectively protected from oxidation through PDMS encapsulation. Based on the comprehensive comparison of material characteristics, this work proposes a synergistic composite structure consisting of a microstructured PDMS upper layer and an AgNWs/paper substrate lower layer. The rationally designed structure integrates the deformation amplification capability of PDMS and the high-efficiency conductive network of AgNWs, effectively avoiding the inherent limitations of individual materials. Consequently, the fabricated sensor achieves a favorable combination of broad pressure detection range and high sensitivity.

Polydimethylsiloxane (PDMS) [13] is a flexible insulating material with excellent chemical and thermal stability, as well as a low elastic modulus, making it ideal for fabricating flexible substrates. Silver nanomaterials, due to their high electrical conductivity and good structural stability, serve as useful modification materials [14]. Most reported pressure sensors with microstructures exhibit high sensitivity only within a very narrow pressure range [15,16,17]. For example, Wang et al. used petals as templates to replicate microstructures in PDMS and then sputtered a layer of zinc oxide nanoparticles to form a piezoresistive dielectric layer; however, the sensor achieved a sensitivity of only 0.28 kPa^−1^ within the 0–2 kPa pressure range [18]. Zhang et al. fabricated a bilayer piezoresistive flexible sensor with dual-height cylindrical microstructures using spin-coated carboxyl-modified multi-walled carbon nanotube (PDMS/MWCNTs-COOH) composite materials, but its sensitivity was merely 1.774 kPa^−1^ in the 0–0.5 kPa pressure range [19]. To advance the large-scale application and mass production of flexible pressure sensors and electronic skins, developing low-cost fabrication processes that enable devices with both high sensitivity and wide pressure response ranges remains a key challenge hindering the industrialization of this technology [20].

Sensors based on carbon nanotubes (CNTs) may have higher baseline resistance, leading to reduced sensitivity; liquid metals exhibit lower intrinsic sensitivity to pressure, limiting their pressure-sensing performance [21]; and hybrid composite materials involve more complex fabrication processes and higher costs [22]. Silver nanowires (AgNWs), in contrast, offer advantages such as high electrical conductivity, excellent mechanical flexibility, good solution processability, and mature synthesis methods. AgNW networks can establish effective conductive pathways at low percolation thresholds, and their one-dimensional morphology enables efficient stress transmission and induces strain-dependent resistance changes. Compared to CNTs, AgNWs typically exhibit lower contact resistance between wire junctions; compared to liquid metals, AgNWs are easier to process and demonstrate better compatibility with paper-based substrates.

To develop a flexible tactile sensor capable of achieving high sensitivity across a wide pressure response range, this study employs silver nanowire-modified toilet paper as the sensing layer and structured polydimethylsiloxane (PDMS) microstructures as the sensitivity-enhancing layer, successfully fabricating a resistive-type flexible tactile sensor. The sensor demonstrates performance in physiological signal detection, featuring high sensitivity, fast response, and stable long-term stability over a broad pressure range. It shows potential for applications in wearable physiological monitoring, intelligent sensing, and multimodal perception, offering an approach for the development and application of paper-based resistive flexible tactile sensors.

## 2. Experimental Section

### 2.1. Materials

Polydimethylsiloxane (PDMS, Sylgard 184, Dow Corning, Midland, MI, USA), consisting of base polymer and curing agent. Silver nanowires (AgNWs, Xianfeng Nanomaterial Technology Co., Ltd., Nanjing, China). Dishwashing liquid (White Cat Co., Ltd., Shanghai, China). 75% ethanol (Shandong Lierkang Disinfection Technology Co., Ltd., Dezhou, China). Epoxy resin AB adhesive. Other experimental materials include toilet paper, filter paper, A4 paper, cotton soft paper, rice paper, and cardstock. The silver nanowires used in this experiment have a diameter of 30 nm, length of 20 μm, concentration of 0.5 mg/mL, and a loading density of approximately 0.15 mg/cm^2^. The paper sheets used are approximately 1.5 cm long, 1 cm wide, and 1 mm thick. The spacing between PDMS microstructures is about 0.5 cm.

### 2.2. Device Fabrication

Use a 4 × 4-inch silicon wafer as the base. Mix the polydimethylsiloxane (PDMS) substrate with the curing agent in a 10:1 mass ratio, stir thoroughly, and then degas under vacuum to remove the bubbles in the mixture. Spin-coat the PDMS mixture onto the silicon wafer array using a spin-coater (XLB-RC-100T, Star Lab Instrument, Jinan, China) and perform thermal curing. Then, peel off to obtain the PDMS template. Prepare a solution of 75% ethanol and dishwashing liquid in a volume ratio of 75:25 (mass ratio 10:1). Immerse the rigid template in this ethanol-dish soap mixture for about 8 h to facilitate the release of PDMS in the subsequent steps. Add additional PDMS mixture to the PDMS template and perform curing before peeling off to obtain the PDMS component of the sensor. Subsequently, deposit silver nanowires and salt particles on the replicated structured PDMS surface; the salt particles serve as additional conductive bridges or electric field concentration points, providing a new and more sensitive pressure response channel for electron transmission. Coat the silver nanowires on the surface of toilet paper, then uniformly heat it using a hot plate (BY2015, BYA Bangyuan Technology, Dongguan, China) to enhance the adhesion between the nanowires and the fiber substrate, forming a stable conductive network. Due to the high conductivity and flexibility of the silver nanowires, they can be embedded or covered on the surface of toilet paper to form conductive channels [23,24]. Subsequently, copper wires were affixed to both ends of the toilet paper/AgNWs sensing layer using conductive silver paste (Huaxin Electronics, Dongguan, China), followed by curing at 60 °C for 30 min to form stable ohmic contacts. The entire device was then encapsulated with silicone rubber to ensure mechanical durability during testing.

**Figure 1:**
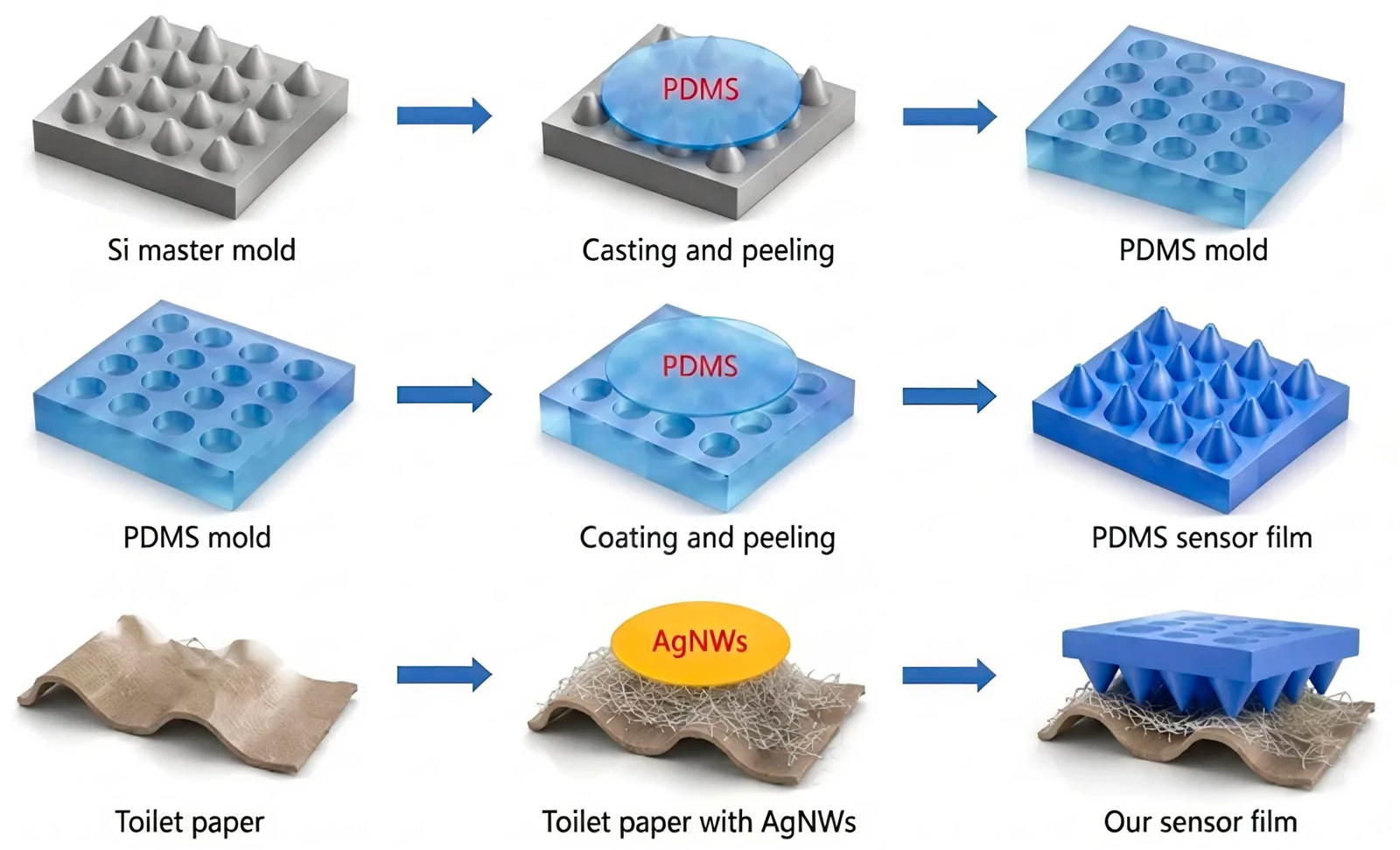
Fabrication process of the sensor.

The PDMS mixture was spin-coated onto the silicon wafer at 1000 rpm for 30 s using a spin-coater (XLB-RC-100T, Star Lab Instrument, Jinan, China), followed by thermal curing at 80 °C for 2 h. The pyramidal microstructures on the PDMS surface had a base width of approximately 100 μm and a height of approximately 50 μm, with a center-to-center spacing of about 0.5 cm. The AgNW suspension (0.5 mg/mL in ethanol) was deposited onto the toilet paper substrate by drop-casting at a loading density of approximately 0.15 mg/cm^2^, followed by drying on a hot plate (BY2015, BYA Bangyuan Technology, Dongguan, China) at 60 °C for 10 min to remove the solvent and enhance adhesion between the nanowires and paper fibers. The complete fabrication workflow of the piezoresistive tactile sensor is illustrated in Figure 1.

## 3. Results and Discussion

### 3.1. Material Characterization

**Figure 2:**
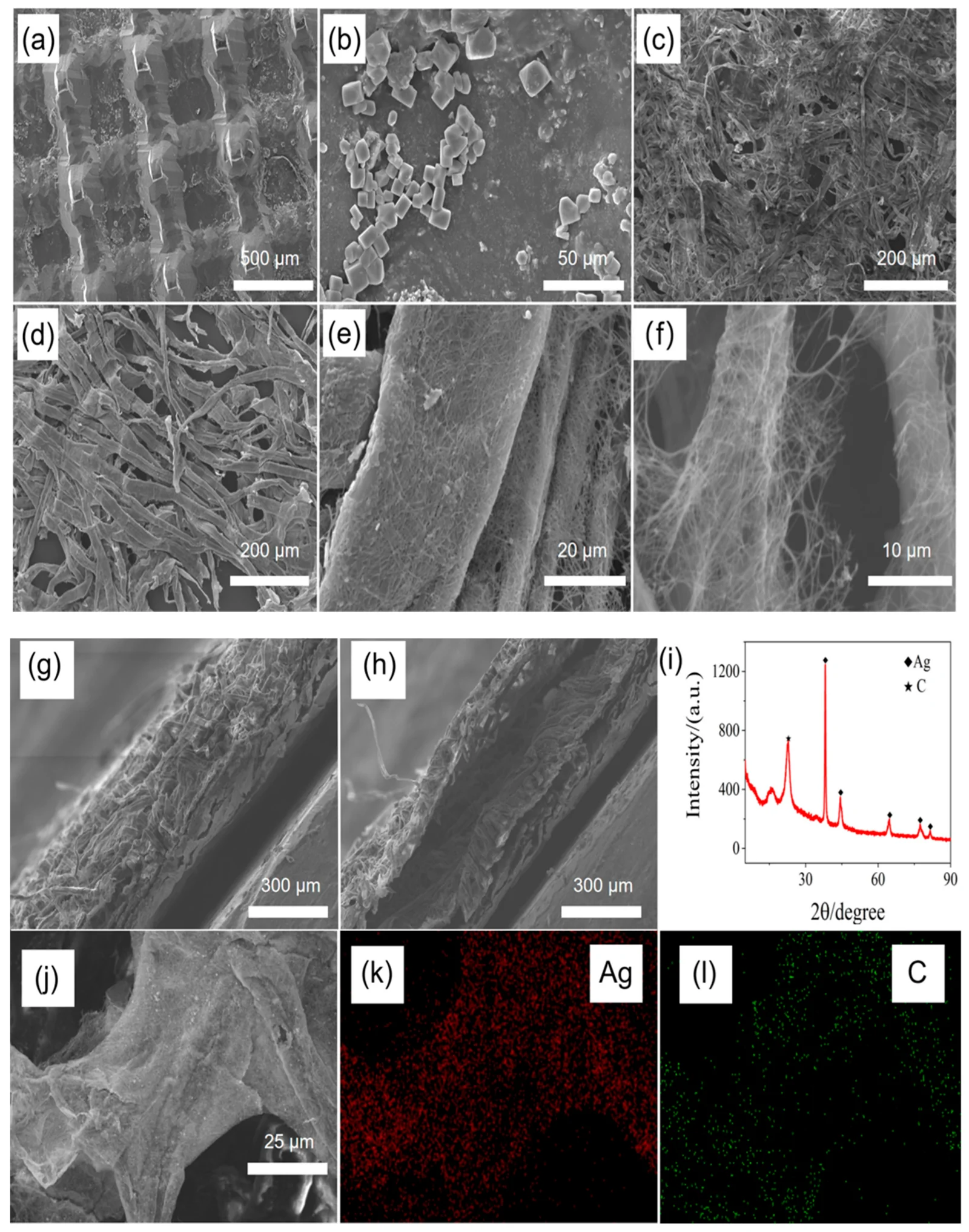
Morphological and structural characterization of PDMS and toilet paper/AgNWs. (**a**) Overview of the engineered PDMS (500 μm); (**b**) salt particles inside the PDMS (50 μm); (**c**) overview of toilet paper (200 μm); (**d**) overlapping fibers of toilet paper (20 μm); (**e**) layered fibers of toilet paper (500 μm); (**f**) interlaced silver nanowires (10 μm); (**g**,**h**) cross-sectional SEM image (300 μm); (**i**) X-ray diffraction (XRD) spectra; (**j**–**l**) elemental mapping (Ag, C).

The morphology of the dense toilet paper/silver nanowire composite film and the processed PDMS structure as well as salt particles was characterized using scanning electron microscopy (SEM, Quattro S, Thermo Fisher Scientific, Hillsboro, OR, USA),as shown in Figure 2. The XRD and SEM-EDX tests were conducted on the sensors to characterize the types and contents of the internal elements. Sample specimens were coated with gold using an SBC-12 ion sputtering coater (KYKY Technology, Beijing, China) to enhance their conductivity. The surface morphology of the films was examined using a biological microscope (Nikon, Tokyo, Japan). The sensing performance of the piezoresistive sensor was tested using a Keithley 2400 high-precision digital multimeter (Keithley Instruments, Cleveland, OH, USA) controlled by a computer, with supporting equipment including a single-axis servo electric cylinder, a DP832 programmable DC power supply (Siglent Technologies, Shenzhen, China), and a pressure testing machine (Aedburg, Jinan, China). All tests were conducted via LabVIEW 2023 (National Instruments, Austin, TX, USA) under an input voltage of 1 V.

### 3.2. Performance Testing of the Sensor

**Figure 3:**
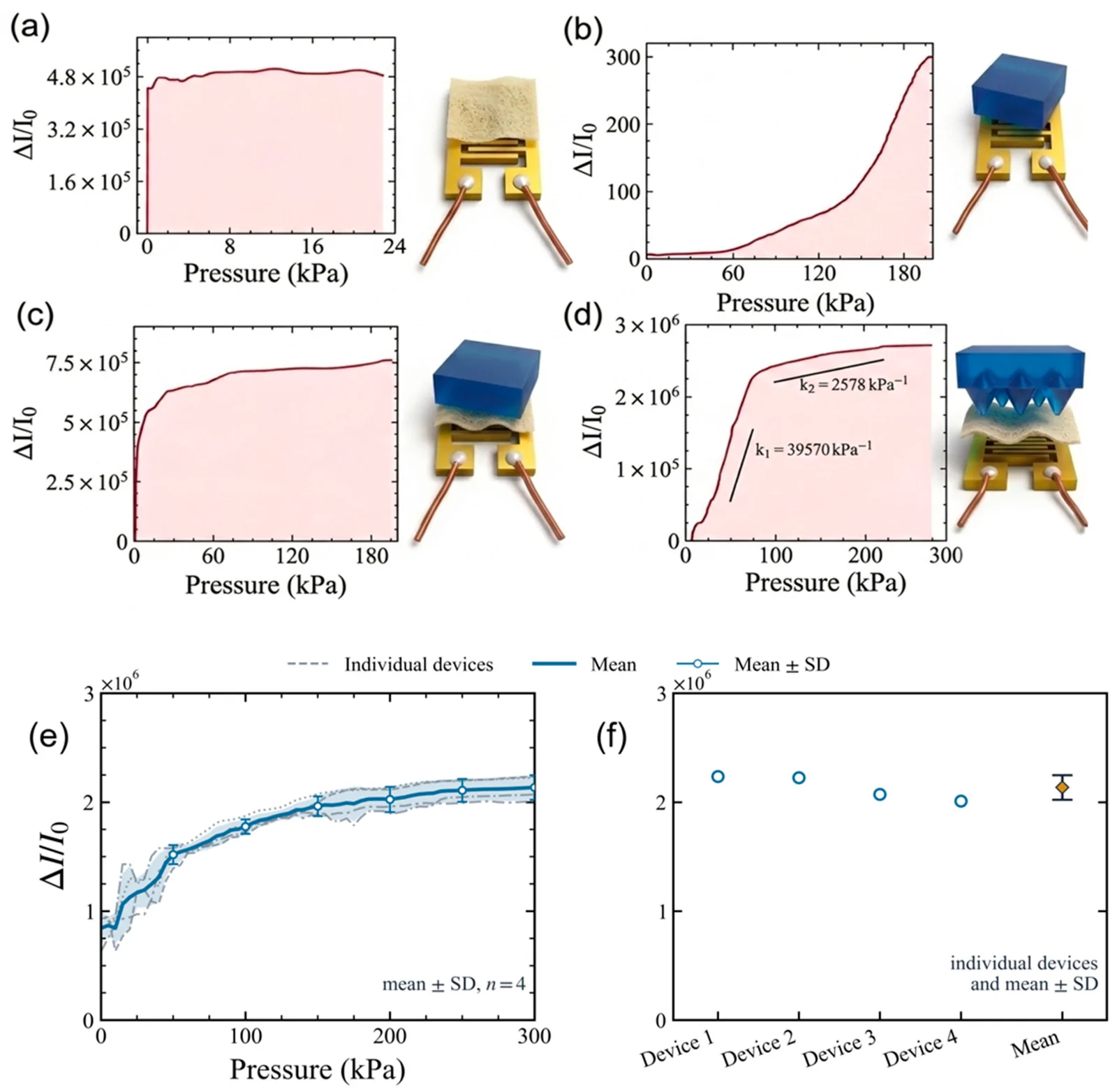

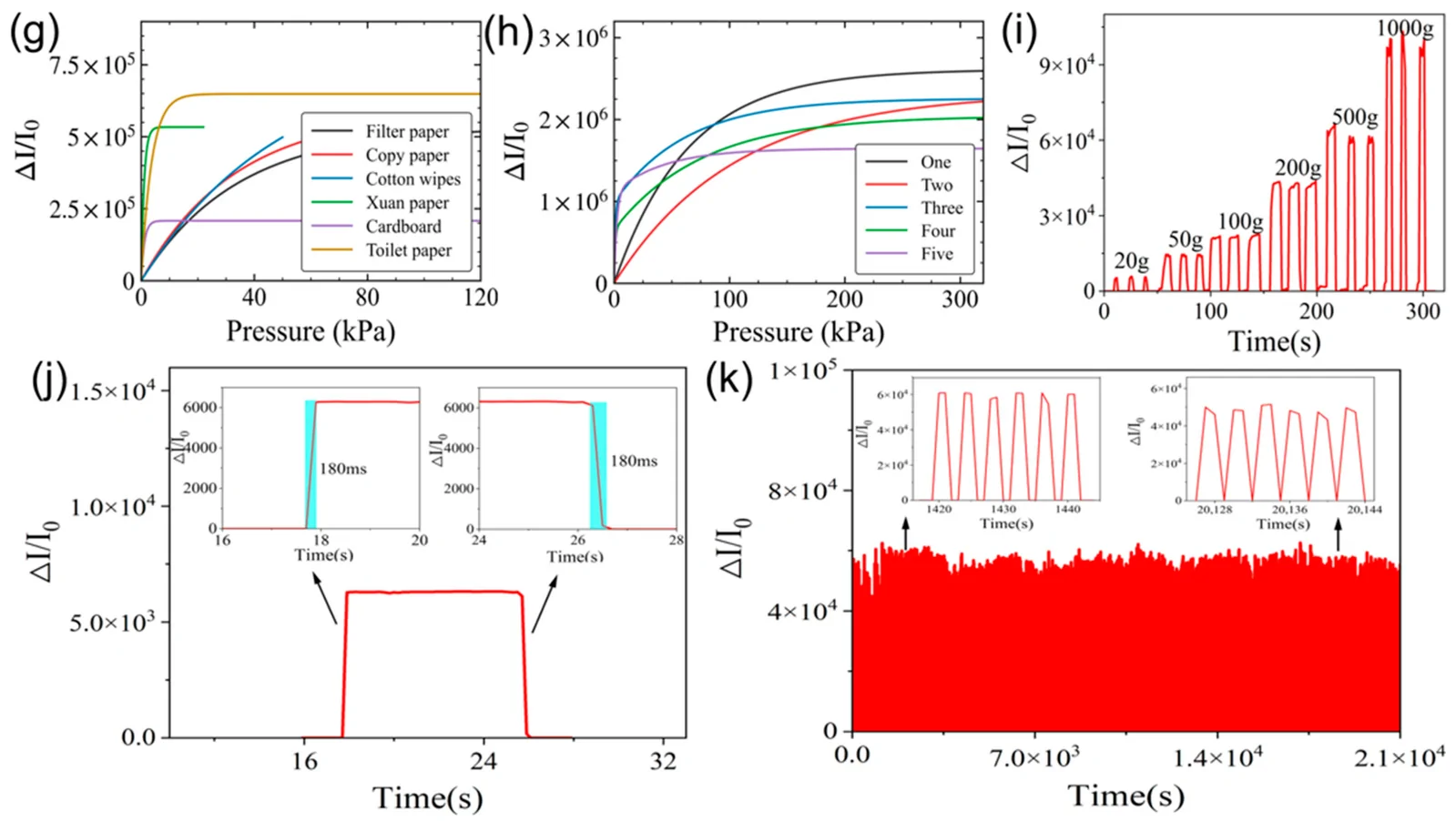
Performance comparison of different device configurations. (**a**) Device A (toilet paper/AgNWs only); (**b**) Device B (PDMS/AgNWs only); (**c**) Device C (smooth PDMS + toilet paper/AgNWs); (**d**) Device D (engineered PDMS + 1-layer toilet paper/AgNWs); (**e**,**f**) phenogram of multiple independently fabricated devices; (**g**) sensitivity of different paper types; (**h**) sensitivity of Devices D–H with 1 to 5 layers of toilet paper (1L = D, 2L = E, 3L = F, 4L = G, 5L = H); (**i**) response under different weights; (**j**) response and recovery time; (**k**) cyclic stability of Device D.

Comparing Device A (Figure 3a) with Device B (Figure 3b) shows that the toilet paper/AgNWs composite exhibits higher conductivity than the PDMS/AgNWs composite. Moreover, toilet paper offers advantages such as low cost and practicality; therefore, this study selects toilet paper over PDMS as the substrate for the sensor. Comparing Device A, Device C (Figure 3c), and Device D (Figure 3d) reveals that Device D (engineered PDMS + toilet paper) achieves the highest sensitivity among all configurations. Device C exhibits limited contact performance under low pressure, resulting in minimal deformation and weak signal changes, ultimately leading to low sensitivity. In contrast, the pyramidal protrusions of Device D concentrate stress at their tips, which compress the toilet paper fibers and enable closer contact between the silver nanowires, thereby increasing conductive pathways. This increases the number of contact points within the conductive network, reduces the resistance, and thus increases sensitivity. In addition, in order to enhance the reliability of the experiment, we use multiple independently fabricated devices (Figure 3e,f).

This study develops a sensor performance testing system and employs OriginPro 2024b to visualize the measured data. The system consists of a pressure testing machine, a servo motor, a high-precision multimeter, and a DC power supply. Sensitivity is the key parameter for evaluating sensor response performance. Performance tests were conducted on various paper-based substrates, including toilet paper, filter paper, A4 paper, cotton soft paper, Xuan paper, and cardstock. Paper samples were placed on the pressure testing machine platform, and after stabilization, the machine was slowly lowered while simultaneously recording current variation data. Experimental results show that toilet paper exhibits the highest sensitivity curve and the highest peak sensitivity. Moreover, compared to other types of paper, toilet paper demonstrates smaller test fluctuations and greater stability (see Figure 3g).

Next, we investigated the effect of paper layer number using Devices D–H (Figure 3h). The peak sensitivity decreases progressively from Device D (1 layer) to Device H (5 layers). The single-layer paper (Device D) is the thinnest and allows almost all silver nanowires to remain on the surface, forming the most complete conductive network. As the overall paper thickness increases, internal pores and fiber layers begin to play a role. Some silver nanowires penetrate the interface between layers, causing a few breakpoints in the surface conductive network and leading to a decrease in conductivity. When the paper is too thick, a large number of silver nanowires become trapped deep within the paper and cannot form effective conductive pathways on the surface. Achieving good conductivity in this case would require a substantial increase in the amount of silver nanowires, raising the cost. Therefore, we used a single, thinnest layer of toilet paper as the sensor substrate.

Next, we analyzed the sensitivity of Device D (engineered PDMS + single-layer toilet paper). Sensitivity (S) of the piezoresistive sensor is defined as the slope of the relative current change versus applied pressure, S = (ΔI/I_0_)/ΔP, where I_0_ is the baseline current measured at 0 kPa under a constant voltage of 1 V. The sensor exhibits two distinct linear regions: the first region is 39,570 kPa^−1^ (6.85–73.26 kPa),the minimum detectable pressure is about 6 kPa. During this stage, the sensor responds rapidly, with a sensitivity of 39,570 kPa^−1^ in this region. The second region is 2578 kPa^−1^ (73.26–273.96 kPa), At this stage, the conductive network is close to saturated and the sensitivity decreases (Figure 3d).

Device D, a dual-layer heterostructure, cleverly combines the advantages of polydimethylsiloxane (PDMS) and toilet paper. The toilet paper/AgNWs layer itself exhibits conductivity, serving as a stable, highly conductive substrate that enhances sensitivity. The surface-modified PDMS layer protects the internal AgNW network within the toilet paper. Moreover, compared to pure toilet paper-based designs (Device A), this composite material demonstrates better bending resistance and flexibility, while the pyramid-like microstructure of PDMS further improves the sensor’s sensitivity.

This study used standard weights of 20 g, 50 g, 100 g, 200 g, 500 g, and 1000 g to test the sensor’s sensitivity (see Figure 3i). The results indicate that the sensor has a response time of 180 ms and a recovery time of 180 ms as well (see Figure 3j), demonstrating its ability to quickly detect and sensitively respond to external pressure changes. During both pressure loading and unloading, the sensor’s response signals remained largely consistent, indicating low hysteresis characteristics. To evaluate the sensor’s long-term cyclic stability and repeatability under continuous pressure loading, approximately 10,000 cycles were conducted (see Figure 3k). The results further show that the sensor signal remained essentially unchanged throughout all repeated cycles, confirming its stability. The testing frequency during this cyclic test was approximately 0.5 Hz, with an applied pressure of about 39.2 kPa per cycle. The observed signal drift primarily arises from the viscoelastic creep of the PDMS microstructure and the microscopic rearrangement of the silver nanowire network under sustained pressure. Under constant pressure loading, paper fibers undergo slight compression while the PDMS exhibits stress relaxation, gradually increasing the effective contact area between silver nanowires and ultimately leading to a slow decay in resistance. This phenomenon is an inherent characteristic of flexible sensors fabricated on soft substrates, determined by the viscoelastic properties of the constituent materials. Regarding cyclic stability, after 10,000 loading/unloading cycles, the sensor retains over 80% of its initial resistance. This high retention rate indicates that the silver nanowire network maintains structural integrity without significant breakage or irreversible delamination from the PDMS/paper substrate. The fatigue resistance is mainly attributed to the stable interfacial adhesion and mechanical robustness of the silver nanowires, confirming the device’s suitability for long-term operation.

In addition, we preliminarily examined the sensor performance under different environmental conditions, including sunny and rainy days as well as noon and evening periods; the observed variations in output current were minimal, suggesting reasonable short-term environmental stability for daily wearable applications.

**Figure 4:**
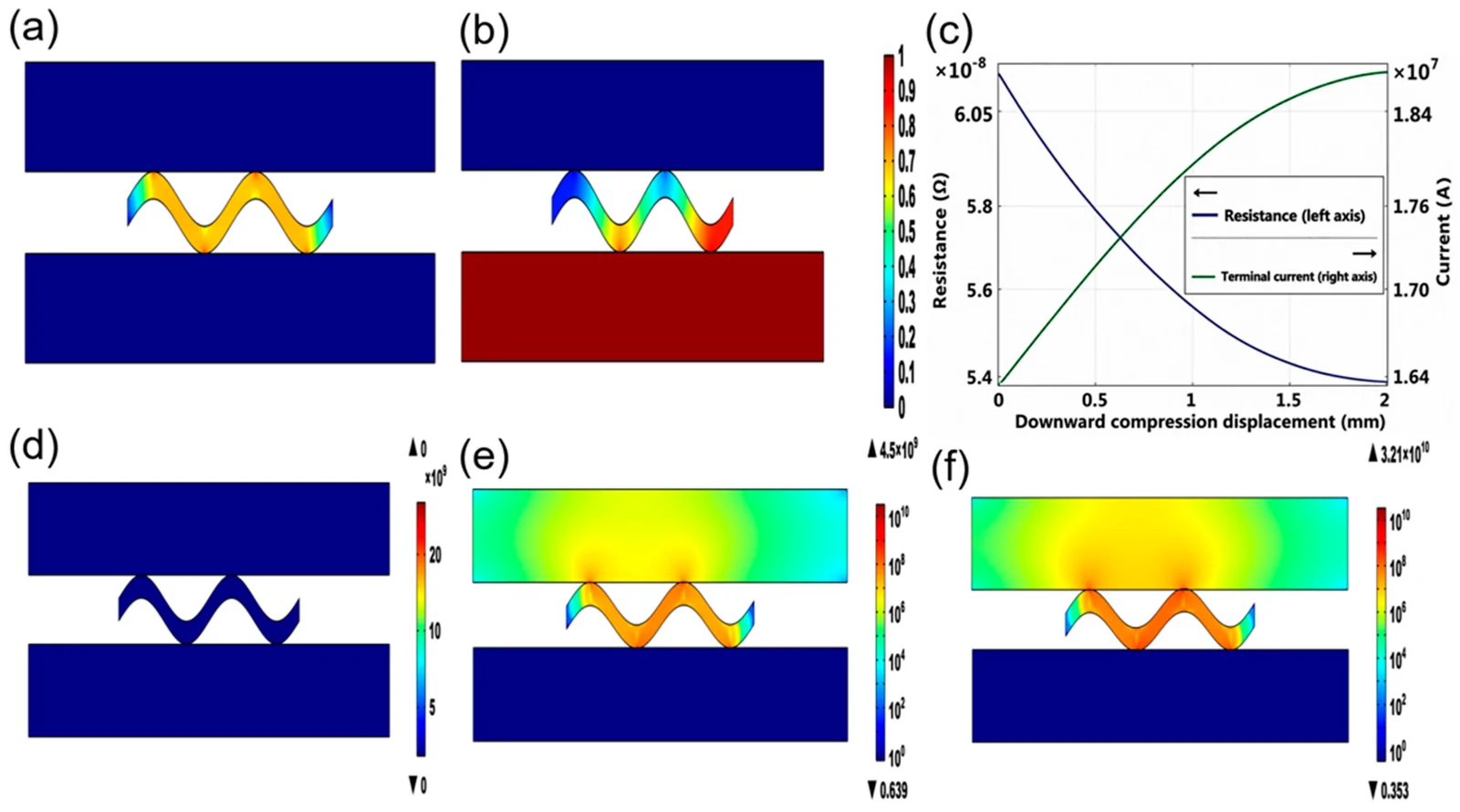
Contact area evolution and finite element simulation. (**a**) Electric field distribution; (**b**) electric potential distribution; (**c**) resistance–current variation curve; (**d**) the pressure distribution and deformation when the downward compression displacement is 0 mm; (**e**) the pressure distribution and deformation when the downward compression displacement is 1 mm; (**f**) the pressure distribution and deformation when the downward compression displacement is 2 mm.

Compared to recently reported piezoresistive sensors based on silver nanowire/polydimethylsiloxane (AgNWs/PDMS), the device fabricated in this study achieves higher sensitivity and a wider operational range. As shown in the contact area evolution process in Figure 4 and the finite element simulation, as the downward compression displacement increases, the contact area expands, the resistance decreases, and thus the current further increases, thereby enhancing the sensitivity of the sensor. This higher performance stems from the synergistic effect between the structured PDMS pyramid microarchitecture and the conformal AgNW network on a toilet paper substrate: the structure enables rapid changes in contact area during compression and efficiently establishes conductive pathways (Table 1) [25,26,27,28,29,30].

### 3.3. The Application of the Sensor

**Figure 5:**
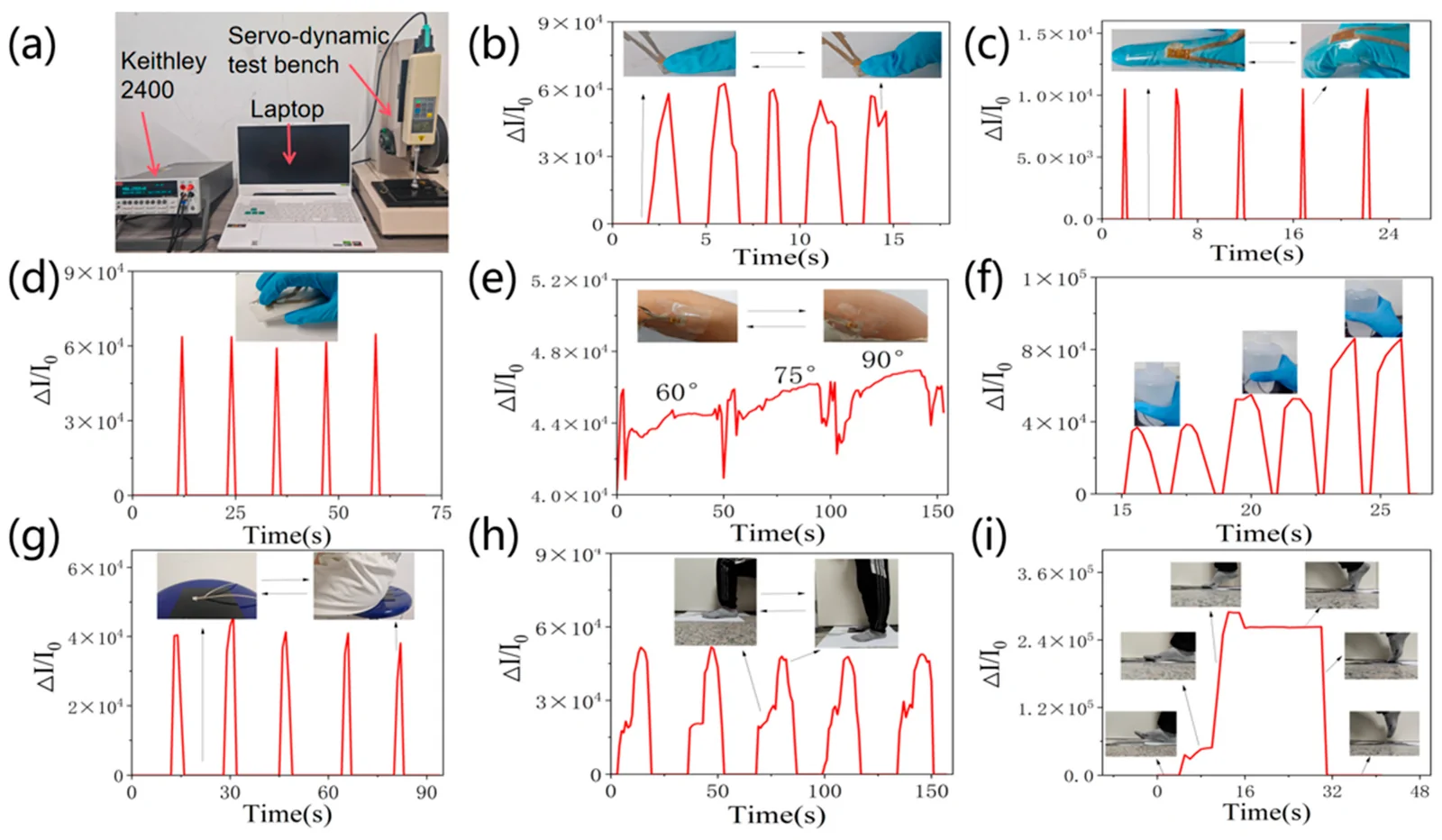
Demonstration of the engineered PDMS/toilet paper-based sensor in human applications. (**a**) Testing setup; (**b**) finger pressing; (**c**) finger bending; (**d**) hand pressing on a mouse; (**e**) elbow bending; (**f**) schematic of hand holding a flask; (**g**) seat cushion; (**h**) standing and sitting; (**i**) walking.

Similar to recently reported implantable smart textiles for motion monitoring, our sensor demonstrates performance in detecting human motion signals [31]. The pressure-strain sensor made of modified PDMS and toilet paper has significant application potential in motion activity monitoring and health assessment. Moreover, when integrated with the host computer, this sensor can achieve precise monitoring of human movements, for signal detection and output. Keithley 2400 SourceMeter (Keithley Instruments, Cleveland, OH, USA) is used for electrical signal acquisition. Laptop is used for program control and data processing. Servo-dynamic test bench is used for applying quantitative pressure to sensors (Figure 5a). Tests were conducted on the fingertip (Figure 5b,d), finger joints (Figure 5c), and elbow joint (Figure 5e). Results indicate that the sensor consistently and stably outputs strong current response signals during finger pressing, finger joint bending, and elbow movements at various angles.

The sensors were also installed on the side walls of the beaker. When the beaker was empty, half-filled with water, or completely filled with water, they were held by hand respectively to monitor the changes in the current signal (Figure 5f). The experimental results showed that as the amount of water in the beaker increased, the pressure exerted by the hand on the sensor gradually increased, and the resulting current also rose accordingly. These experimental results further confirm that this sensor has sensitive response and stable performance.

We also applied this sensor to seat cushions (Figure 5g) and the walking monitoring system (Figure 5h,i). As shown in Figure 5h,i, when the foot touches the sensor and lands, the current rises to a certain level; when the foot is about to lift and move forward, the center of gravity shifts toward the toe, causing the current to rise sharply to a larger value; when the toe completely leaves the sensor, the current drops to zero. This indicates that the sensor can sensitively detect the human state.

## 4. Conclusions

In summary, we have developed a flexible tactile sensor with a wide pressure range and high sensitivity. The design principle is to encapsulate a microstructure-sensitive PDMS layer on top of a toilet paper/silver nanowire substrate. By leveraging the structural advantages of Device D (engineered PDMS microstructure) and its synergistic effect with the toilet paper/AgNWs layer, the sensitivity of the device has been improved. This flexible tactile sensor exhibits a wide-pressure detection capability (6.85–273.96 kPa), high sensitivity (39,570 kPa^−1^), fast response/recovery time (180 ms), and good stability and cycle repeatability (approximately 10,000 cycles). The sensor achieves higher sensitivity and wider operational range. It is equivalent to developing a high-performance paper-based engineered platform at a relatively low cost. With these features, we have demonstrated the various applications of this sensor on the human body, proving its promising potential in wearable biosensing and related fields.

## Figures and Tables

**Table 1:** Comparison of sensing performance between the proposed piezoresistive sensor and other reported flexible pressure sensors.

Things	Sensitivity	Range	Response Time (ms)	LOD (kPa)	Reference
PDMS/ZnO	0.28	0–2 kPa	<30	0.16	[18]
PDMS/MWCNTs-COOH	1.774	0–0.5 kPa	N/A	N/A	[19]
MXene/SWCNT	2.6	5.1 Pa	56	0.0051	[25]
TPU@MWCNTS (DGM)	69.8	0–300 kPa	5	N/A	[26]
PDMS/CB/MWCNTs	14.61	0–70 kPa	320	N/A	[27]
PVA/AgNWs/TPU	47.65	1–238 kPa	N/A	N/A	[28]
MXene/MWCNTs-PDMS	9041.8	0–1228.75 kPa	N/A	N/A	[29]
PDMS micro-pyramid/AgNWs	-	0–1600 kPa	40	N/A	[30]
PDMS/toilet paper/AgNWs	39,570	6.85–273.96 kPa	180	6.85	Our work

Note: N/A denotes that the corresponding parameter was not reported in the cited references.

## Data Availability

All the data supporting the conclusions of this study are included in the main body of the paper. Further inquiries can be directed to the corresponding author(s).
